# Exploring E-Cigarette Use Among Indonesian Youth: Prevalence, Determinants and Policy Implications

**DOI:** 10.1007/s10900-025-01442-0

**Published:** 2025-02-09

**Authors:** Razan Wibowo, Tobias Weinmann, Dennis Nowak, Yayi Suryo Prabandari

**Affiliations:** 1 Institute and Clinic for Occupational, Social and Environmental Medicine, LMU University Hospital, LMU Munich, Munich, Germany; 2 Institute for Medical Information Processing, Biometry and Epidemiology (IBE), LMU Munich, Munich, Germany; 3 Pettenkoffer School of Public Health, Munich, Germany; 4 Comprehensive Pneumology Centre (CPC) Munich, Member of German Centre for Lung Research (DZL), Munich, Germany; 5 Department of Health Behaviour, Environmental, and Social Medicine (HBES), Faculty of Medicine, Public Health, and Nursing, Universitas Gadjah Mada, Yogyakarta, Indonesia

**Keywords:** e-Cigarette, Indonesia, Youth smoking, Tobacco control, Public health

## Abstract

The growing popularity of electronic cigarettes (e-cigarettes) among Indonesian youth presents a new public health challenge in a country with one of the highest tobacco burdens globally. While tobacco control regulations have been implemented, e-cigarettes remain largely unregulated, raising concerns about their health impacts and youth appeal. This study explores the prevalence, determinants and perceptions of e-cigarette use among high school and university students in Indonesia, providing critical insights to inform effective policymaking. A cross-sectional online-based survey was conducted in 2019 among 158 students aged 15–30 years across 17 provinces in Indonesia. Data on tobacco use behaviours, socioeconomic background, social influences and health perceptions were collected. Descriptive and inferential statistical analyses identified key determinants of e-cigarette use. Among the participants, 36.2% reported ever smoking, and 65.2% of these had tried e-cigarettes. Key determinants of e-cigarette use included male gender, urban residency, peer smoking and social acceptance of smoking (all *p* < 0.05). Notably, participants commonly perceived e-cigarettes as less harmful than combustible cigarettes and helpful for smoking cessation, despite conflicting scientific evidence. Overall, e-cigarette use is prevalent among Indonesian youth, driven by social and environmental factors, as well as misconceptions about safety. Strengthened regulations and targeted public health campaigns are essential to mitigate the health risks posed by e-cigarettes and enhance tobacco control efforts.

**Supplementary Information:**

The online version contains supplementary material available at 10.1007/s10900-025-01442-0.

## Implications

This study provides critical insights into the prevalence and patterns of e-cigarette use among Indonesian youth, highlighting key determinants such as social influences and misconceptions about e-cigarettes. The widespread belief that e-cigarettes are safer than combustible cigarettes and can aid in smoking cessation presents a significant public health concern. These findings highlight the need for a comprehensive evaluation of Indonesia´s current tobacco control regulations, with a focus on strengthening public awareness campaigns and enforcement practices. Effective policy changes are essential to mitigate the health risks of e-cigarettes and improve overall tobacco control efforts.

## Introduction

### Tobacco use in Indonesia

Indonesia, with an estimated population exceeding 267 million in 2018 [1], faces a significant public health challenge due to high rates of tobacco consumption [2–5]. Despite evidence of its harmful effects, smoking prevalence remains persistently high [6], driven by factors such as affordability, aggressive marketing by the tobacco industry, and weak enforcement of tobacco control measures [7–9]. Community-based surveys, including the Indonesian Family Life Survey and the 2018 Global Adult Tobacco Survey (GATS), reported an alarming trend, with smoking rates among adults increasing from 54% in 1995 to approximately 67% in 2015, despite a slight decline to 63% in 2018 [3],RAND [4, 10], with men dominating this increase [11], as 2 out of 3 Indonesian men smoke [12].

Adolescents and young adults represent a particularly vulnerable demographic. In fact, an increase in smoking prevalence was observed in those aged 10–18 years, rising from 7.2% in 2013 to 9.1% in 2018 [3]. Given that youth compromise approximately 18% of Indonesia´s population [13], these trends foreshadow an increased burden of non-communicable diseases at an early age [14–16] and economic costs linked to tobacco-related illnesses [17]. Addressing this issue requires evidence-based policy interventions targeting youth tobacco consumption [6, 7].

### Tobacco Control Regulations and Emerging Alternative Products

Although Indonesia has yet to ratify the WHO Framework Convention on Tobacco Control (Tobacco Control Laws [18, 19]), the country has implemented partial tobacco control measures in 2008 and has received technical assistance through the enforcement of Tobacco Control Regulation (TCR) (European [20, 21]). These include smoke-free areas, packaging and labelling regulations, and regulation of tobacco advertising, promotion, and sponsorship (Ministry of Health Regulation No. 56 of 2017 amends some of the provisions of Regulation No. 28 of 2013) [6],European [20],Tobacco Control Laws [18, 22]. A well-known example of TCR implementation in Indonesia is the adoption of cigarette warning labels, which contain some critical (pictorial) information about the harmful effects of smoking. Nevertheless, enforcement remains inconsistent, limiting their impact [22, 23].

In response to growing awareness of the harms of combustible cigarettes, e-cigarettes and other electronic nicotine delivery systems have emerged as alternative products. Globally, their used has increased significantly [24, 25],Jessica K [26]. Surveys conducted by the International Tobacco Control Policy Evaluation Project between 2009 and 2013 reported varying prevalence rates of e-cigarette use, ranging from 14% in Malaysia to lower estimates in Global North countries such as the US (6%), the UK (4%), the Netherlands (3%), and Canada (1%) [25]. In Indonesia, GATS data indicate a significant increase in e-cigarette use, rising from 0.3% in 2015 to 11.9% in 2018 [3, 4, 27].

While some studies suggest e-cigarettes may serve as harm-reduction tools [28–33], other raise concerns about their potential to sustain nicotine dependence and act as a gateway to smoking, particularly among youth [34, 35],J.-F. [36],J. F. [37, 38]. Young people are drawn to e-cigarettes due to perception of a ‘‘less harmful effect’’ [25, 28, 30, 32], appealing flavours [39, 40],J. K. [41], curiosity [39, 40, 42, 43] and the overall trend of growing popularity [44]. However, evidence also suggests that prior smoking experience is a significant predictor of e-cigarette use [29, 32, 45–47].

Despite the rising popularity of e-cigarettes, research on their prevalence, determinants and perceptions, particularly among adolescents and young adults in Indonesia, remains scarce. Based on these considerations, the present study aimed to fill the gap by assessing e-cigarette use among high school and university students in Indonesia. It seeks to examine patterns of use, underlying perceptions and influencing factors to provide insights for evidence-based policymaking.

## Methods

### Study Design and Sample

This study employed a quantitative cross-sectional survey, conducted between July and August 2019. Convenience sampling was used to recruit high school and university students from 17 provinces in Indonesia, aiming for geographic diversity. Participants provided consent and completed a customised online-based questionnaire (see Measures). The required sample size, calculated for 90% confidence at a 10% significance level, was determined for a minimum of 101 participants. Ultimately, 158 participants took part in the study. Eligibility criteria for participation required individuals to be active high school or university students and aged at least 15 years (equivalent to 10th grade), as this is the age at which the consumption of tobacco products can be freely observed [12, 48]. The upper age limit was set at 30 years, reflecting the typical age of workforce entry in Indonesia (signifying the end of student life) (*The Work Permit (KITAS) Regulations in Indonesia (No. 16/2015) and Its October 2015 Update (No. 35/2015), as Well as the Latest Presidential Regulation (No. 20/2018) for The Use of Foreign Worker*[49], 2018). Recruitment included outreach through university social media platforms (e.g., Facebook or Twitter).

### Measures

#### Demographic and Socioeconomic Characteristics

The online questionnaire collected data on demographic and socioeconomic characteristics such as (1) gender, (2) age by the time of survey, (3) residence (urban/rural), (4) education level (high school or equivalent degree, some vocational degree, as well as undergraduate and graduate degree or higher), (5) ethnicity (Javanese vs. other; specified), (6) parental education (separated between father and mother, three categories: low (no education to primary school), medium (junior to senior high school), and high (vocational diploma/undergraduate to graduate/postgraduate)), and (7) monthly income (including monthly pocket money: < Rp. 1,000,000 (~ < USD 65) (low); Rp. 1,000,000—5,000,000 (~ USD 65 – USD 320) (medium); and > Rp. 5,000,000 (~ > USD 320) (high)) [13].

#### Tobacco Use Behaviours

Tobacco consumption was assessed using initial questions adapted from the 2019 Youth Risk Behaviour Survey: (1) "Have you ever tried smoking cigarettes, even 1 or 2 puffs?" [28, 29] and (2) "Even if you have never tried smoking cigarettes, have you ever tried using other tobacco products (e.g., small cigars, water pipes, vape, chewing tobacco)?" [29]. Participants who responded with 'never tried' to both questions were classified as ‘never smokers’. Those who responded with ‘ever tried’ to at least one of these two initial questions were classified as ‘ever smokers’. Further questions covered smoking/consumption frequency, age at initiation, recent use of e-cigarettes and motives of use.

#### Tobacco-Related Social Environment

Social factors have been strongly associated with cigarette smoking [15],M. [50, 51] and more recently with e-cigarette use among adolescents [28, 33, 52],M. [16, 50]. Questions based on test items employed in previous studies, were used to assess peer and household influences, including: (1) peer smoking: "How many of your 4 closest friends smoke cigarettes?" (0–4 friends); (2) peer acceptance of smoking: "How would your best friends behave towards you if you smoked cigarettes?" (5-point Likert scale, ranging from "very unfriendly" to "very friendly"); and (3) exposure to smoking at home: "Does anyone who lives with you now use cigarettes?" (yes/no; and if yes, please specify relationship to you) [52],M. [50, 51].

#### Health Literacy and Perceptions

Participants responded to items on tobacco knowledge, behaviour and perceptions using item clusters developed from previous studies: (1) knowledge: e.g. ‘I know that smoking in general has a negative effect on my health’ (yes/no/not sure); (2) behaviour: e.g., ‘In the past 12 months, have you ever tried to quit smoking/using all tobacco-related products (including combustible cigarettes, e-cigarettes, vape, shisha, chewing tobacco)?" (I am not a smoker/yes, and I have not used any tobacco products in the last 12 months/yes, but it is difficult for me/no); and (3) perception: e.g., "I think that smoking or using alternative products (e.g., vape, chewing tobacco) can help people to quit smoking regular cigarettes" (5-point Likert scale, consisting of strongly disagree, disagree, neutral, agree and strongly agree) (M. [50, 51]).

#### Survey Validation

All survey instruments used were translated into Indonesian and developed following the WHO recommendation for translation and adaptation [53–55]. Experts in medical and social sciences selected, translated and reviewed its content (see **Supplementary Figure 1**). A feasibility test with 20 randomly selected respondents evaluated statistical biases. Test results were discussed with the team to identify any potential statistical biases. Validity was confirmed using Pearson product-moment correlation and internal consistency (reliability) was evaluated using Cronbach’s alpha coefficient (> 0.70 for both high school and university students).

### Data Handling and Statistical Analysis

Data cleaning was carried out to screen eligible participants based on the inclusion criteria. Dropout participants, defined as those who stopped participating after completing the socioeconomic part, were excluded. The final sample consisted of 127 participants. Excel (Version 11.0, Microsoft Corporation®, Redmond, USA) was used for initial data processing, followed by comprehensive data analysis performed using Statistical Programme for Social Sciences (SPSS®, Version 23.0, IBM, Armonk, USA). Descriptive statistics described sample characteristics. The Pearson’s chi-squared tests analysed associations between categorical variables, such as demographic factors and e-cigarette use. Alternative methods, such as logistic regression, were considered but not applied due to the study´s sample size and cross-sectional design. The alpha (α) level was set at 0.05 for all statistical tests. All p-values were two-tailed.

## Results

### Participant Characteristics

Of the 158 participants assessed, 127 were eligible and included in the study. Participants were dichotomised into two age groups: 15–18 years (48.8%) and 19–30 years (51.2%), assuming the average age of leaving high school and entering university. The majority resided in urban areas (59.8%). Most participants were high school students (43.3%), primarily from Java, although mostly identified themselves as non-Javanese.

Regarding the tobacco-related social environment, peer smoking was prevalent, with many participants reported having friends who smoked. In addition, 39.5% were exposed to smoking behaviour at home, with fathers or male siblings being the most common smokers. Table 1 provides a detailed overview of the participants’ socioeconomic and socioenvironmental characteristics.

**Table 1:** Socioeconomic and social-environmental factors of the study population (N = 127)

Variable	N (%)	*Missing (n)*
**Socioeconomic**		
**Sex**		*0*
Female	73 (57.5)	
Male	54 (42.5)	
**Age group**		*0*
15–18	62 (48.8)	
19–30	65 (51.2)	
**Residential area**		*0*
Rural	51 (40.2)	
Urban	76 (59.8)	
**Ethnicity**		*0*
Javanese	59 (46.4)	
Others	68 (53.5)	
**Educational background**		*0*
High school	55 (43.3)	
Vocational college	15 (11.8)	
Undergraduate degree	35 (27.6)	
Graduate degree	22 (17.3)	
**Parental Education**		*0*
Father		
Low	11 (8.7)	
Middle	57 (44.9)	
High	59 (46.4)	
Mother		
Low	18 (14.2)	
Middle	53 (41.7)	
High	56 (44.1)	
**Monthly income**		*0*
Low	69 (54.3)	
Middle	33 (26.0)	
High	25 (19.7)	
**Tobacco-related social environment**		
**Peers smoking**		*3*
0	42 (33.9)	
1	22 (17.7)	
2	19 (15.3)	
3	16 (12.9)	
4 (all of them)	25 (20.2)	
**Peers’ acceptance of smoking**		*3*
Very unfriendly	24 (19.4)	
Unfriendly	28 (22.6)	
Neutral	52 (41.9)	
Friendly	11 (8.9)	
Very friendly	9 (7.2)	
**Tobacco consumption by family member / flatmate**		*3*
Yes	49 (39.5)	
No	75 (60.5)	

### Overall Pattern of Tobacco Use

The prevalence of ever-smoking was 36.2% (Table 2). Among ever-smokers, most first tried combustible cigarettes between ages 15–17 (30.4%) or 13–14 (19.6%). However, 43.5% had not smoked a combustible cigarette in the past 30 days.

**Table 2:** The smoking pattern of the study population

Variable	N (%)	*Missing (n)*
General smoking behaviour*		*0*
Ever smoker	46 (36.2)	
Never smoker	81 (63.8)	
**Combustible cigarettes**		
***Age at first use***		
< 8	5 (10.9)	*0*
8–10	8 (17.4)	
11–12	4 (8.7)	
13–14	9 (19.6)	
15–17	14 (30.4)	
> 17	6 (13.0)	
***Day(s) used to smoke***		*0*
No in the past 30 days	20 (43.5)	
1–2	7 (15–2)	
3–5	7 (15.2)	
6–9	0	
10–19	0	
20–29	5 (10.9)	
All 30 days	7 (15.2)	
***Daily number of smoked cigarettes***		*0*
No in the past 30 days	21 (45.6)	
< 1	4 (8.7)	
1	3 (6.5)	
2–5	6 (13.0)	
6–10	3 (6.5)	
11–20	5 (10.9)	
> 20	4 (8.7)	
**e-cigarettes (*N* = 46)**		
e-cigarettes using behaviour*		*0*
Ever users	30 (65.2)	
Never users	16 (34.8)	
***Day(s) used***		*0*
No in the past 30 days	0	
1–2	7 (23.3)	
3–5	0	
6–9	4 (13.3)	
10–19	6 (20.0)	
20–29	5 (16.7)	
All 30 days	8 (26.7)	
***Access to e-cigarettes***		*0*
No in the last 30 days	15 (50.0)	
Bought in stores	3 (10.0)	
Internet	1 (3.3)	
Gave someone else money	0	
Borrowing	5 (16.7)	
Vapor bar/lounge	5 (16.7)	
Stealing	1 (3.3)	
Any other way	0	
***Reason to use other products***		*0*
No in the last 30 days	0	
Less harmful effects	19 (65.5)	
Peers/environmental influence	1 (3.4)	
Desire to quit smoking	5 (17.2)	
Sensation seeking	2 (6.9)	
Any other reason	2 (6.9)	
**Alternative products (*N* = 46)**		
Consumption behaviour*		*0*
Ever users	29 (63.0)	
Never users	17 (37.0)	
***Day(s) used***		*20*
No in the past 30 days	0	
1–2	1 (11.1)	
3–5	0	
6–9	1 (11.1)	
10–19	1 (11.1)	
20–29	2 (22.2)	
All 30 days	4 (44.4)	
***Access***		*1*
No in the past 30 days	18 (64.3)	
Bought in stores	6 (21.4)	
Internet	1 (3.6)	
Gave someone else money	0	
Receiving from others	1 (3.6)	
Stealing	1 (3.6)	
Any other way	1 (3.6)	
***Reason to use other products***		*1*
No in the last 30 days	16 (57.1)	
Less harmful effects	2 (7.1)	
Peers/environmental influence	4 (14.3)	
Desire to quit smoking	2 (7.1)	
Sensation seeking	1 (3.6)	
Any other reason	3 (10.7)	

^*^Initial questions

Of those who smoked combustible cigarettes, 65.2% had also tried e-cigarettes, with vape being the most common product used. In addition, 26.7% reported e-cigarette use within the past 30 days. E-cigarette access was facilitated through borrowing, visiting vape bars/lounges (both 16.7%), or purchasing from convenience stores (10%). Reasons for vaping included perceived reduced harm (65.5%), quitting smoking (17.2%) and sensation seeking (6.9%).

For other alternative products, 63.0% of smoking students identified themselves as ever-users. Products such as glo, chewing tobacco and snuff were most common. Unfortunately, only nine respondents reported the number of days of use, with most reporting use within the past 30 days (44.4%). Access methods varied, with 64.3% reporting no purchases within the last 30 days, while others bought them in shops (21.4%). Motivations included peer influence (14.3%), a desire to quit smoking (7.1%), and other reasons (10.7%).

### Perceptions and Beliefs Regarding E-Cigarette Use

Participants´ awareness of smoking´s negative effects (sub-cluster K1) and of the 2013 tobacco control regulations (sub-cluster K2) was high. Most of them (68.8%) reported taking pictorial warnings on cigarette packs/advertisements seriously (sub-cluster A1). However, 27.9% disagreed that e-cigarettes are less harmful than combustible cigarettes (sub-cluster P1), and 39.3% doubted that e-cigarettes contain fewer harmful ingredients (sub-cluster P4). Regarding participants’ views on the policy regulation of e-cigarette use in Indonesia (sub-cluster P6), 36.9% strongly agreed, and 30.3% agreed with stricter policies. Table 3 shows the detailed distribution of participants’ responses across all clusters of their perceptions and beliefs about e-cigarettes.

**Table 3:** Exploration of participant´s perceptions and beliefs of e-cigarettes (N = 127)

Variable clusters	N (%)	*Missing (n)*
**Knowledge (K)**		
***Negative impact of smoking (K1)***		*5*
No knowledge	0	
Not sure	0	
Yes	122 (100.0)	
***2013 Tobacco Control Regulation (K2)***		*5*
No knowledge	19 (15.6)	
Not sure	19 (15.6)	
Yes	84 (68.8)	
**Attitude (A)**		
***Taking seriously attention to cigarettes pictorial warnings (A1)***		*5*
No	24 (19.7)	
Not sure	14 (15.5)	
Yes	84 (68.8)	
***Trying to quit smoking during the past*** ***12 months (A2)***		*5*
No intention	12 (9.8)	
Yes, but difficult	17 (13.9)	
Yes, and now quit	12 (9.8)	
I am a non-smoker	81 (66.4)	
**Perception (P)**		
***Less harm effect of e-cigarettes (P1)***		*5*
Strongly agree	12 (9.8)	
Agree	20 (16.4)	
Neutral	28 (22.9)	
Disagree	34 (27.9	
Strongly disagree	28 (22.9)	
***e-cigarettes can help to quit smoking (P2)***		*5*
Strongly agree	10 (8.2)	
Agree	15 (12.2)	
Neutral	29 (23.8)	
Disagree	45 (36.9)	
Strongly disagree	23 (18.9)	
***Less addictive effect of e-cigarettes (P3)***		*5*
Strongly agree	11 (9.0)	
Agree	27 (22.1)	
Neutral	35 (28.7)	
Disagree	34 (27.9)	
Strongly disagree	15 (12.3)	
***e-cigarettes do not contain nicotine/TAR (P4)***		*5*
Strongly agree	9 (7.4)	
Agree	16 (13.2)	
Neutral	22 (18.0)	
Disagree	48 (39.3)	
Strongly disagree	27 (22.1)	
***Ability to pay more for less harm effect (P5)***		*5*
Strongly agree	10 (8.2)	
Agree	14 (11.5)	
Neutral	27 (22.1)	
Disagree	33 (27.0)	
Strongly disagree	38 (31.1)	
***Regulation regarding e-cigarettes (P6)***		*5*
Strongly agree	45 (36.9)	
Agree	37 (30.3)	
Neutral	23 (18.9)	
Disagree	7 (5.7)	
Strongly disagree	10 (8.2)	

### Factors Contributing to E-Cigarettes and Other Alternative Products Use

E-cigarette use was significantly higher among smoking male high school/university students (50.0%) than female (15.2%). Urban residency also showed higher usage rates compared to rural counterparts (*p* = 0.021). All three factors clustered in the tobacco-related social environment: peer smoking, peer acceptance of smoking behaviour, and exposure at home were significantly associated with e-cigarette consumption among ever-smokers. For other alternative products, residential area and peer smoking were statistically significant factors influencing use (see Table 4).

**Table 4:** Determinants of e-cigarette and other alternative product use

Variable	e-cigarettes (*N* = 46)			Other alternative products (*N* = 46)		
	**Yes (%)**	**No (%)**	***p*-value**	**Yes (%)**	**No (%)**	***p*-value**
***Sex***			.009**			*.068*
Female	7 (15.2)	9 (19.6)		10 (21.7)	7 (15.2)	
Male	23 (50.0)	7 (15.2)		19 (41.3)	10 (21.7)	
***Age group***			*.079*			*.110*
15–18	11 (23.9)	6 (13.0)		12 (26.1)	9 (19.6)	
19–30	19 (41.3)	10 (21.7)		17 (36.9)	8 (17.4)	
***Residential area***			.021*			.027*
Rural	7 (15.2)	12 (26.1)		5 (3.3)	3 (6.5)	
Urban	23 (50.0)	4 (8.7)		24 (33.3)	14 (30.4)	
***Education***			*.140*			*.568*
High school	10 (21.7)	6 (13.0)		2 (4.3)	5 (10.9)	
Vocational degree	4 (8.7)	3 (6.5)		7 (15.2)	6 (13.0)	
Undergraduate degree	13 (28.3)	5 (10.9)		10 (21.7)	3 (6.5)	
Graduate degree	3 (6.5)	2 (4.3)		10 (21.7)	3 (6.5)	
***Ethnicity***			*.592*			*.757*
Javanese	12 (26.1)	6 (13.0)		14 (30.4)	6 (13.0)	
Other	18 (39.1)	10 (21.7)		15 (32.6)	11 (23.9)	
***Parental education***						
**Father**			*.474*			*.705*
Middle	14 (30.4)	9 (19.6)		16 (34.8)	7 (15.2)	
High	16 (34.8)	7 (15.2)		13 (28.3)	10 (21.7)	
**Mother**			*.578*			*.464*
Low	4 (9.7)	0		0	1 (2.2)	
Middle	11 (23.9)	8 (17.4)		12 (16.7)	6 (13.0)	
High	15 (32.6)	8 (17.4)		17 (20.0)	10 (21.7)	
***Monthly income***			*.367*			*.506*
Low	9 (19.6)	7 (15.2)		6 (13.0)	9 (19.6)	
Middle	9 (19.6)	5 (10.9)		10 (21.7)	5 (10.9)	
High	12 (26.1)	4 (8.7)		13 (28.3)	3 (6.5)	
***Peer smoking***			.001**			.007*
0 (no smoker)	0	6 (13.0)		0	2 (4.3)	
1	3 (6.5)	5 (10.9)		3 (6.5)	4 (8.7)	
2	4 (8.7)	3 (6.5)		4 (8.7)	6 (13.0)	
3	6 (13.0)	2 (3.2)		7 (15.2)	1 (2.2)	
4 (all of them)	17 (36.9)	0		15 (32.6)	4 (8.7)	
***Peer acceptance of smoking***			.033*			*.463*
Very unfriendly	0	2 (4.3)		3 (6.5)	1 (2.2)	
Unfriendly	3 (6.5)	3 (6.5)		7 (15.2)	4 (8.7)	
Neutral	17 (36.9)	8 (17.4)		8 (17.4)	10 (21.7)	
Friendly	7 (15.2)	3 (6.5)		6 (13.0)	1 (2.2)	
Very friendly	3 (6.5)	0		5 (10.9)	1 (2.2)	
***Tobacco consumption of family member / flatmate***			.002**			*.346*
Yes	21 (45.6	8 (17.4)		15 (32.6)	10 (21.7)	
No	9 (19.6)	8 (17.4)		14 (30.4)	7 (15.2)	

Note: ^***^: *p* < 0.001; **: *p* < 0.01; *: *p* < 0.05

## Discussion

The present study revealed that 36.2% of the surveyed 127 Indonesian high school and university students were smokers of varying frequencies. Among these, nearly two-thirds had ever used e-cigarettes, suggesting that previous cigarette smoking is a key predictor of e-cigarette use. This finding aligns consistently with prior research confirming associations between tobacco consumption and the adoption of alternative products, such as e-cigarettes [24, 28, 32, 33, 45–47, 52],M. [50, 51]. Consistently, the 2018 GATS reported a notable increase in e-cigarette use in Indonesia since 2015 [3, 4, 27].

Key determinants of e-cigarette use found in this study included male gender, urban residence and peer influence, reflecting previous findings (J.-F. [36],J. F. [37, 56])[28, 29, 33, 52],M. [50, 51]. Men, often early adopters of new technologies, are more likely to experiment with e-cigarettes [57] (J.-F. [36],J. F. [37, 56]). In addition, urban settings further facilitate easier access to both the products and related advertisements [33],M. [50]. In contrast, female e-cigarette use remain less defined, despite increasing female smoking rates in many countries [58–60]. Cultural taboos, for example, may discourage women from smoking in Indonesia [61].

Social determinants, such as peer smoking, peer acceptance of smoking and tobacco exposure at home, also contributed to e-cigarette use in our study population. Despite more than half of participants disagreeing the notion that e-cigarettes are less harmful than combustible cigarettes or aid in smoking cessation, these misconceptions were still prevalent among e-cigarette users. This pattern echoes the findings of Chapman et al. (2014), who reported that e-cigarettes are often perceived as substitutes rather than cessation aids [62].

Misconceptions about e-cigarettes being less harmful and more cost-effective than combustible cigarettes in terms of health-related economic burden also emerged. Kozlowski et al. [63] highlighted that such perceptions may influence uptake [63]. However, case reports have documented toxicity in e-cigarette ingredients, leading to respiratory allergic reactions [64–66], emphasizing the need for accurate risk communication.

Most participants demonstrated awareness of the harmful effects of combustible cigarettes, reflecting increased health consciousness. Consistent with Palipudi et al. [67], higher education levels were linked to greater awareness of e-cigarettes [67]. Applying the Health Belief Model [68–70], the study identified three interrelated drivers of e-cigarette use: (1) knowledge of smoking risks, (2) perceived health severity, and (3) beliefs influenced by advertising that frames e-cigarettes as safer alternatives, which further complicating the public´s understanding of their risks[32, 34].

The existing TCR in Indonesia, including cigarette warning labels, shows promise but face enforcement challenges [61]. Establishing clearer regulations and standards for e-cigarettes, combined with rigorous surveillance, are essential to address health risks and curb tobacco use in Indonesia.

Given the widespread use of e-cigarettes and other heat-not-burn products, future research should assess their long-term health impacts. To the best of our knowledge, although marketed as healthier alternatives to combustible cigarettes, e-cigarette use is not without risk, albeit lower than those of conventional tobacco products due to reduced level of carcinogenic chemicals [64–66]. Strengthening public awareness and implementing stricter policies can support comprehensive tobacco control efforts in Indonesia.

## Limitations

This study has some limitations. First, the data were primarily collected in Java, which, although the most populous island in Indonesia, may limit the diversity of participants. However, given that Java represents approximately 54% of the country´s population [13], the findings may still be broadly generalizable to the national proportion. Second, the cross-sectional approach employed in this study, as in many others, limits the ability to draw causal inferences. While associations between variables can be identified, the study cannot establish causality [71],B. [72, 73]. Additionally, the use of an online-based questionnaire may introduce reporting bias, particularly in adolescents and young adults who might underreport their tobacco consumption due to social desirability. There is also potential selection bias, as smokers may have been more likely to participate in this study, given the topic´s relevance, while non-smokers may have had less interest.

Overall, despite these limitations, the study provides a valuable profile of e-cigarette and alternative product use among Indonesian high school and university students. It could also offer a basis for future research on this issue and provide a framework to guide decision-making.

## Conclusion

In summary, e-cigarette use among Indonesian youth remains a significant public health challenge, particularly due to social and environmental determinants such as peer influence and urban accessibility. While many participants were aware of the harms of combustible cigarettes, misconceptions about e-cigarettes’ safety and efficacy in smoking cessation persist. This study underscores the urgent need for targeted public health interventions, strengthened regulations, and awareness campaigns to address these misconceptions and mitigate health risks. Integrating comprehensive e-cigarette regulations into existing tobacco control frameworks is essential to curtail nicotine addiction and its broader public health implications.

## supplementary material

Below is the link to the electronic supplementary material.

Supplementary Material (31KB)
