# Supplementary material for: Exploring E-Cigarette Use Among Indonesian Youth: Prevalence, Determinants and Policy Implications
Source: J Community Health. 2025 Feb 9;50(4):585–95. doi: 10.1007/s10900-025-01442-0 (PMC12301275; doi:10.1007/s10900-025-01442-0)
Supplement: Supplementary file 1 — Supplementary Material (31KB) [file 10900_2025_1442_MOESM1_ESM.docx]

**Supplementary Figure**

**Supplementary Figure 1:** The validation process of the survey instrument. This figure illustrates the steps taken to adapt and validate the survey instrument, including comparisons made by the study team and cultural adaptations to ensure its relevance for the Indonesian context.

DATA COLECTION AND ANALYSIS

The instrument was administered by a native Indonesian speaker. All study team, the first and last authors, reached a consensus after comparing and adapting the instrument to ensure that its meaning was appropriate for Indonesian culture.

PROOFREADING

FEASIBILITY STUDY

The pre-final version was tested with a panel of 20 respondents. Short interviews were conducted to explore potential difficulties in understanding and misinterpretations of survey items.

BACKWARD TRANSLATION

The first-reconciled forward version of the survey instrument was translated back into English by another member of study team. This person is fluent in both English and the target language and did not have access to the original version of the instrument.

The source language (English) was translated into the target language (Indonesian) by the first author and a study team, both of whom are native Indonesian speakers and fluent in the source language.

FORWARD TRANSLATION
